# Proteasome inhibitors – molecular basis and current perspectives in multiple myeloma

**DOI:** 10.1111/jcmm.12279

**Published:** 2014-04-08

**Authors:** Lenka Kubiczkova, Ludek Pour, Lenka Sedlarikova, Roman Hajek, Sabina Sevcikova

**Affiliations:** a Babak Myeloma Group, Department of Pathological Physiology, Faculty of Medicine, Masaryk UniversityBrno, Czech Republic; b Department of Clinical Hematology, University Hospital BrnoBrno, Czech Republic; c Department of Internal Medicine - Hematology and Oncology, University Hospital BrnoBrno, Czech Republic; d Department of Hematooncology, Faculty of Medicine University of Ostrava and University Hospital OstravaOstrava, Czech Republic

**Keywords:** multiple myeloma, new-generation proteasome inhibitors, bortezomib

## Abstract

Inhibition of proteasome, a proteolytic complex responsible for the degradation of ubiquitinated proteins, has emerged as a powerful strategy for treatment of multiple myeloma (MM), a plasma cell malignancy. First-in-class agent, bortezomib, has demonstrated great positive therapeutic efficacy in MM, both in pre-clinical and in clinical studies. However, despite its high efficiency, a large proportion of patients do not achieve sufficient clinical response. Therefore, the development of a second-generation of proteasome inhibitors (PIs) with improved pharmacological properties was needed. Recently, several of these new agents have been introduced into clinics including carfilzomib, marizomib and ixazomib. Further, new orally administered second-generation PI oprozomib is being investigated. This review provides an overview of main mechanisms of action of PIs in MM, focusing on the ongoing development and progress of novel anti-proteasome therapeutics.

- Introduction
- Proteasome inhibitors in multiple myeloma
- Bortezomib
  Mechanism of action
  NF-κB pathway
  Apoptotic pathway
  Cell cycle and migration
  Effect of bortezomib on MM side population
  Bortezomib and microRNA
  Further effects
- Second-generation of proteasome inhibitors
  Carfilzomib
  Ixazomib
  Marizomib
- Novel proteasome inhibitors
  Oprozomib
  Delanzomib
- Mechanism of resistance and cross-resistance of Pls
- Induction of neuropathy
- Proteasome inhibitors in other diseases
- Conclusion

## Introduction

The degradation of cellular proteins is a tightly regulated and complex process that plays a central role in regulating cellular function and maintaining homoeostasis in every eukaryotic cell [1]. The ubiquitin-proteasome pathway (UPP) represents the major pathway for intracellular protein degradation. More than 80% of cellular proteins are degraded through this pathway, including those involved in the regulation of numerous cellular and physiological functions, such as cell cycle, apoptosis, transcription, DNA repair, protein quality control and antigens [2,3].

Proteasome as a new cell structure was described by Harris group in the beginning of 1970s as a hollow cylinder and single-torus proteins [4]. Later, it was elucidated that the function of proteasome is an ATP-dependent degradation of intracellular proteins, and its specificity is determined by interaction only with such proteins that are labelled by polyubiquitin chain or contain a specific amino acid sequence [1,5]. For this important discovery, Ciechanover, Hershko and Rose received the Nobel Prize in chemistry in 2004.

**Figure 1:**
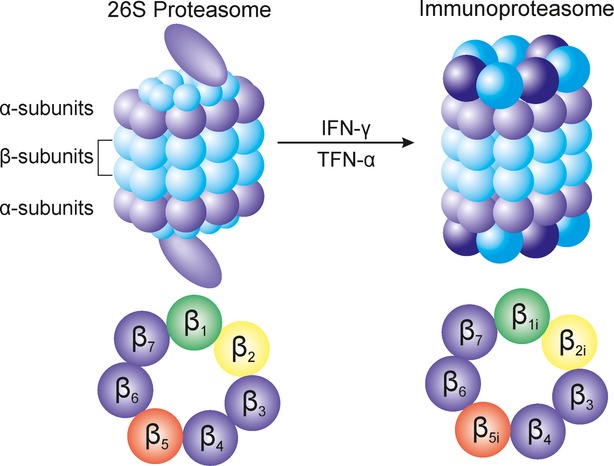
Structure of 26S proteasome and immunoproteasome and its catalytic subunits. 26S proteasome consists of regulatory particle and proteolytic core region containing four subunits (arranged as α-β-β-α). In cells of hematopoietic origin, various stimuli, such as interferon (IFN)-γ and tumour necrosis factor (TNF)-α induce synthesis of immunoproteasome. Arrangement of proteolytically active subunits: β1 (β1i) – caspase-like subunit, β2 (β2i) – trypsin-like subunit, β5 (β5i) – chymotrypsin-like subunit of proteasome and immunoproteasome is displayed.

Human 26S proteasome is formed by 20S proteolytic core region and 19S regulatory particle. 20S proteasome is an abundant, barrel-shaped molecule consisting of four highly homologous rings that enclose a central catalytic chamber with proteolytic active sites. Each of the rings contains seven subunits α and β, which are arranged one above the other in the order of α-β-β-α (Fig.1). While the outer two α-rings surround a small opening through which only denatured polypeptide substrates may pass, two central β-rings contain multiple proteolytic sites that function together in protein degradation [6,7]. Each of these two β rings comprises three proteolytic sites - β1 (caspase-like, C-L), β2 (trypsin-like, T-L) and β5 (chymotrypsin-like, CT-L) [8,9]. Exposing cells to few stimuli, such as interferon-γ, tumour necrosis factor-α (TNF-α) and bacterial lipopolysaccharides, induces the synthesis of other catalytic subunits that are together incorporated into alternative proteasome form – immunoproteasome, which is preferentially expressed in cells of lymphoid origin and plays a role in major histocompatibility complex class I antigen presentation and other constitutive proteolytic activities [10–12]. In the 20S immunoproteasome (i20S), proteolytically active subunits β1, β2 and β5 are substituted by their equivalents β1i (LMP2) β2i (MECL-1) and β5i (LMP7; Fig.1) [13]. Although proteasome contains multiple catalytic sites, to inhibit its function at the constitutive or immunoproteasome level, it is sufficient to block only the β5/LMP7 subunit (CT-L) [14,15]. Proteins intended for degradation are incorrectly folded proteins and proteins with short half-life and mostly regulatory function that are being cut into oligopeptide chains with an average length of 8–12 amino acids [16,17].

## Proteasome inhibitors in multiple myeloma

It has become evident that defects within the UPP pathway are associated with a number of diseases, including cancer; thus, inhibitors of this pathway should prevent malignant cells from proliferation [18,19]. The biggest group of proteasome inhibitors (PIs) is short peptides containing covalently attached pharmacophore – a group of atoms that binds to the catalytic sites of proteasome and thus prevents proper proteasome function [20]. Inhibition of proteasome is particularly useful for the treatment of multiple myeloma (MM), a haematological malignancy caused by malignant transformation of B-lymphocytes into pathological clonal plasma cells (PCs) that accumulate in the bone marrow (BM) and secrete high amounts of monoclonal immunoglobulin (Ig) [21]. As both normal and malignant PCs are highly secretory cells, they require a well-developed endoplasmic reticulum (ER), expansion of secretory apparatus and production of chaperone proteins that ensure proper Ig translation and folding [22]. A stress signalling pathway called the unfolded protein response (UPR) ensures that the PCs can handle the proper folding of proteins and prevent the aggregation of accumulating misfolded proteins. These proteins are then transported out of the ER and degraded by proteasome [23,24]. It was shown that treatment of MM cells with PIs results in the accumulation of misfolded Ig within the ER, because of inhibition of proteasome function [25]. Such stress activates the UPR pathway, which is mediated by activation or translational repression of several transcription factors, such as XBP-1, ATF6 and PERK/eIF2α [25,26]. Generally, UPR allows the cell to survive reversible environmental conditions, such as chemical insult or nutrient deprivation. However, during prolonged stress caused by PIs, UPR activation leads to cell cycle arrest [27] and induction of apoptosis [28]. PIs initiate UPR leading to apoptosis preferentially in cells with high Ig production; thus, partial inhibition of proteasome *in vivo*, which is not toxic to patients' normal cells, is sufficient to kill MM PCs [29].

**Figure 2:**
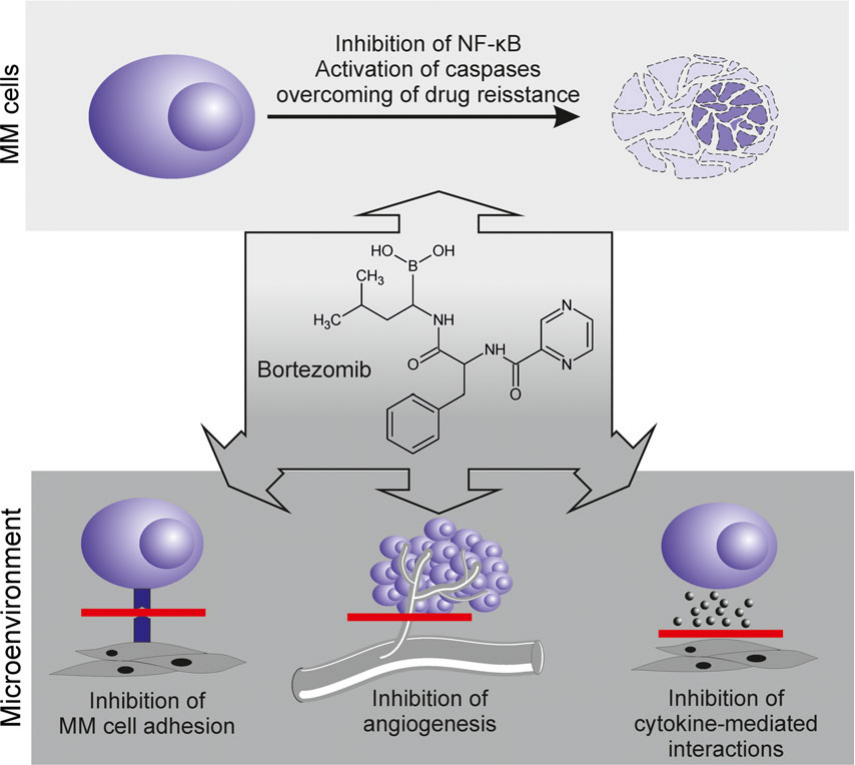
Effects of proteasome inhibitors (PIs; bortezomib) on multiple myeloma (MM) cells and bone marrow (BM) microenvironment. Bortezomib affects MM cell survival and signalling pathways eventually leading to apoptosis. Also, bortezomib influences surroundings of MM cells as it inhibits adhesion of MM cells to BM microenvironment, angiogenesis and cytokine-mediated interactions.

Further, the therapeutic success of PIs in MM relies on their pleiotropic effects, which decrease both growth and survival of MM cells and the interaction between MM cells and BM microenvironment (MM cells adhesion, formation of new blood vessels and cytokine circuits; Fig.2). Treatment with PIs has been associated with reports of increased bone formation markers and decrease in markers of bone resorption, which are the consequences of enhanced osteoblastogenesis and reduced number of osteoclasts [30,31].

The ability of PIs to kill MM PCs and restore proper bone formation led to their use in clinics as one of the therapeutic approaches. Since the approval of first-class PI bortezomib for the treatment of MM, response rates and median survival of MM patients have considerably improved [32,33]. Further, new-generation PIs, such as carfilzomib, ixazomib, marizomib and oprozomib, are based on different chemical moieties than bortezomib and have modified pharmacologic properties, potentially resulting in better clinical outcome and reduced toxicity for MM patients (Table1). Some of them are currently approved for the treatment of MM, the others are being investigated in multiple ongoing clinical studies (Table2).

**Table 1:** Characteristics of proteasome inhibitors evaluated for multiple myeloma treatment

Inhibitor of proteasome	Active moiety	Proteasome target	Key celullar effects	Binding	References
Bortezomib	Boronate	Preferentially CT-L/LMP7, C-L/LMP2 subunit, less T-L/MECL-1 subunit	NF-κB, caspase-8, 9, p21, p27, p53, Bid and Bax, caveolin-1, p-H3, EZH2, miR-29b, miR-15a	Reversible	[87]
Carfilzomib	Epoxyketone	Preferentially CT-L/LMP7 subunit	Caspases-3, 7, 8 and 9, JNK, eIF2, NOXA	Irreversible	[74]
Marizomib	β-lactone	Preferentially CT-L/LMP7 subunit, T-L/MECL-1 subunit, less C-L/LMP2 subunit	Caspase-8, NF-κB	Irreversible	[119]
Ixazomib	Boronate	Preferentially CT-L/LMP7 subunit, less C-L/LMP2 and T-L/MECL-1 subunit	Caspase-8, 9 and 3, p53, p21, NOXA, PUMA, E2F, cyclin D1 and CDK6, Bip, CHOP, miR-33b	Reversible	[82,87
Oprozomib	Epoxyketone	CT-L/LMP7 subunit	Caspases-8, -9, -3, PARP, JNK, NF-κB	Irreversible	[89,120
Delanzomib	Boronate	CT-L/LMP7 subunit	NF-κB	Reversible	[90]

**Table 2:** Clinical development of the drugs and ongoing pivotal trials in MM (according to myeloma.org and clinicaltrials.gov)

	Bortezomib	Carfilzomib	Marizomib	Ixazomib	Oprozomib	Delanzomib
Stage of development	Phase III	Phase III	Phase I	Phase III	Phase I/II	Phase I/II
Pivotal ongoing trials	Clinical trials for use with transplant, induction, consdolidation and maintenance therapy	Various phase III clinical trials including a trial comparing carfilzomib *versus* bortezomib	**NCT00461045:** Clinical trial of NPI-0052 in patients with relapsed or relapsed/refractory MM	**NCT01850524**: MLN9708 in patients with newly diagnosed MM	**NCT01832727:** Multicentre, open-label study of oprozomib and dexamethasone in patients with relapsed and/or refractory MM	Studies have been terminated
				**NCT01564537**: MLN9708 in relapsed/refractory MM		
Approval	**EMA**: front-line, non-transplant, relapse					
	**FDA:** all settings	**FDA:** relapse	Not approved	Not approved	Not approved	Not approved

## Bortezomib

**Figure 3:**
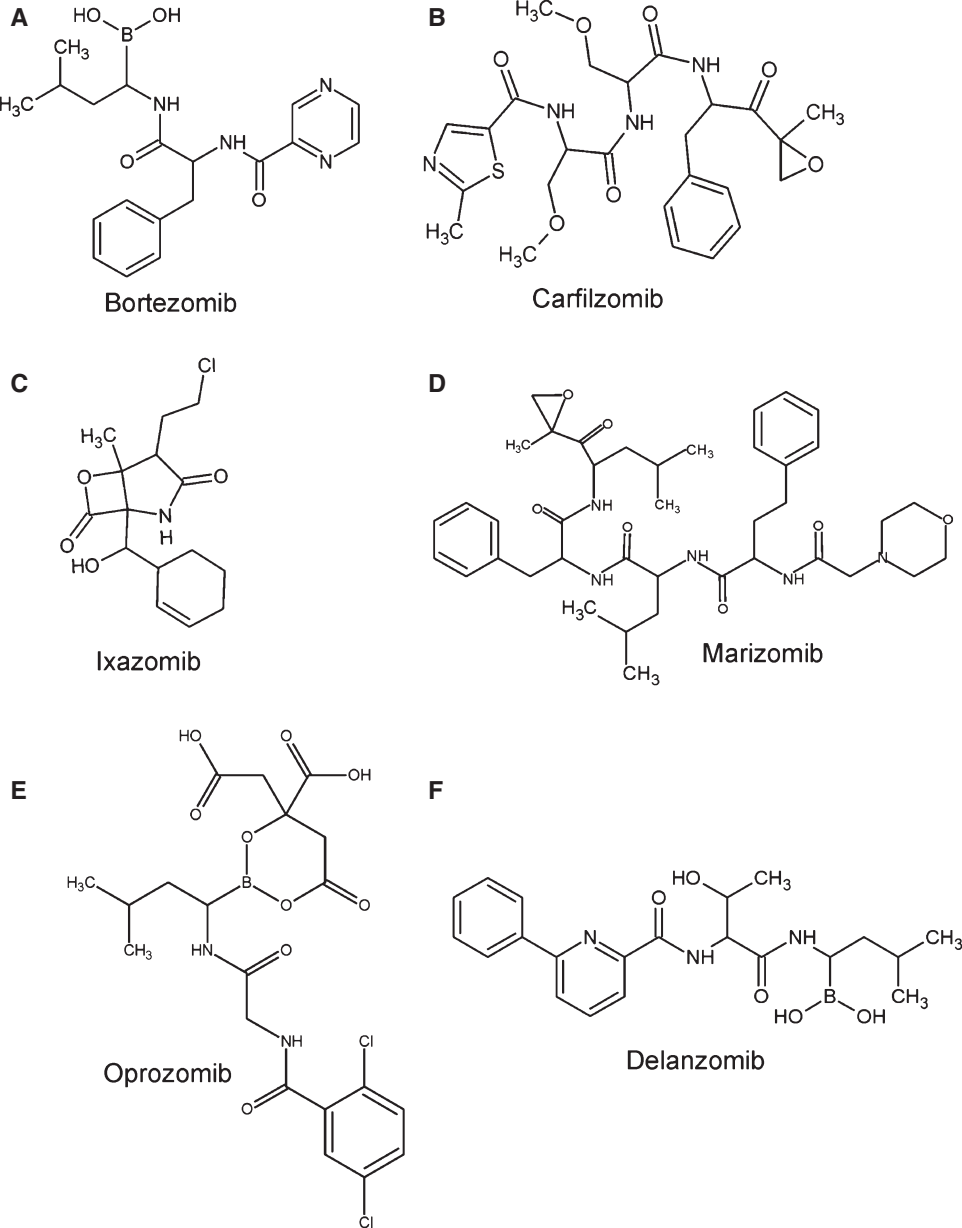
Chemical structures of proteasome inhibitors. (A) Bortezomib, (B) Carfilzomib, (C) Ixazomib, (D) Marizomib, (E) Oprozomib, (F) Delanzomib.

Bortezomib (Velcade), formerly known as PS-341 (Millennium Pharmaceuticals, Cambridge, MA, USA), is the first-class PI that was approved in 2003 for treatment of refractory MM. In 2005, it was approved for treatment of MM patients who had received at least one prior therapy and in 2008 for the treatment of MM patients in first line [34–36]. Further, in 2012, FDA approved subcutaneous administration of bortezomib in all approved indications [37]. Chemically, it is a peptide boronate with molecular formula C19H25BN4O4 (Fig.3A).

Bortezomib was synthesized for the first time in the mid-90s of the last century by Myogenics/ProScript (today Millennium Pharmaceuticals). An *in vitro* study on 60 cancer cell lines confirmed its high specificity, efficiency and oxidative stability [38]. Further, it was shown to potently inhibit cell proliferation in different MM cell lines, either drug sensitive or drug resistant [39]. The first clinical trial using bortezomib in the treatment of haematological malignancies was launched in November 1999. In this study, Orlowski *et al*. showed that low doses of bortezomib, used originally to verify its safety, led to complete remission in a 47-year-old MM patient. Moreover, further eight patients of 11 enrolled in the study showed at least minimal response or stable disease [40]. This result was important as it led to accelerated approval of bortezomib for the treatment of relapsed and refractory MM, after verification in further phases of clinical trials.

### Mechanism of action

Central mechanism of bortezomib function is its covalent binding with high affinity to CT-L (β5) subunit of proteasome or LMP7 subunit of immunoproteasome; however, its binding to C-L (β1) and T-L (β2) subunits with lower affinity has been observed as well [41]. The differences in its affinity are because of different interactions of its side chains with each of the subunits [42]. When bound, bortezomib adopts an anti-parallel β sheet conformation, which is stabilized by direct hydrogen bond between the conserved residues (Gly47N, Thr21N, Thr21O, and Ala49O) of the β-type subunits and main chain atoms of the drug. The actual inhibition is mediated by a pharmacophore group, in this case boronic acid derivative. The boronic acid moiety of the drug ensures increased specificity for the proteasome. The boron atom covalently interacts with the nucleophilic oxygen lone pair of Thr1O^γ^, while Gly47N, stabilizing the oxyanion hole, is hydrogen-bridged to one of the acidic boronate hydroxyl groups. The tetrahedral boronate adduct is further stabilized by a second acidic boronate hydroxyl moiety, which hydrogen-bridges the N-terminal threonine amine atom, functioning as a catalytic proton acceptor. Then, the resulting adduct is characterized by a low degree of dissociation, and therefore remains stable for several hours, even if it is a reversible reaction [42].

Today, the mechanism of action and molecular targets of bortezomib are well characterized. The downstream biological effects of proteasome inhibition are multifactorial, with direct effects on both MM cells and MM cell microenvironment, and key signalling pathways influenced by bortezomib are described further in this review.

### NF-κB pathway

The initial rationale to use bortezomib in cancer was its inhibitory effect on inflammation-associated transcription factor, nuclear factor-κB (NF-κB) through stabilization of its inhibitor I-κB [43]. NF-κB not only regulates various immune and inflammatory responses, but it is also involved in several tumour-related processes, such as suppression of apoptosis and induction of angiogenesis, proliferation and migration. NF-κB is present in the cytoplasm as an inactive complex with its inhibitor I-κB and is activated by proteasomal degradation of I-κB [44]. As PIs inhibit function of proteasome, they prevent degradation of I-κB, subsequent translocation of NF-κB to the nucleus and binding to the promoters of target genes (such as anti-apoptotic genes, interleukin-6 *etc*.) [45]. It was elucidated that MM cell adhesion to BM stromal cells (BMSCs) induces NF-κB-dependent up-regulation of interleukin-6 (IL-6) expression by BMSCs [46,47]. Therefore, inhibition of NF-κB could prevent IL-6 expression, which triggers terminal differentiation of normal B-cells and stimulates growth of MM cells [48].

Although pre-clinical and clinical studies with bortezomib showed down-regulation of transcriptional targets of NF-κB, further studies demonstrated that bortezomib is able to induce I-κB down-regulation that occurred at a transcriptional or post-transcriptional level in MM cell lines [49]. This study further showed that effect of bortezomib is cell dependent, as it triggered NF-κB activation *via* the canonical pathway, associated with down-regulation of I-κB in peripheral blood mononuclear cells, but significantly inhibited NF-κB in BMSCs. Further, it was demonstrated that bortezomib promotes non-proteasomal degradation of I-κB, as it activates two upstream NF-κB-activating kinases (RIP2 and IKKβ) and therefore is able to directly or indirectly (*via* RIP2) activate IKKβ, which subsequently phosphorylates I-κB leading to its degradation [49]. A hypothesis that instead of I-κB stabilization, bortezomib induces I-κB degradation was confirmed by a later study in which I-κB degradation by bortezomib occurred early before induction of apoptosis and could be prevented by calpain inhibitors. Therefore, in the presence of calpain inhibitors, the apoptosis-inducing activity of bortezomib was dramatically enhanced [50].

As bortezomib inhibits inducible NF-κB activity in MM cells, but enhances constitutive NF-κB activity *via* activation of the canonical pathway, bortezomib-induced cytotoxicity cannot be completely attributed to inhibition of canonical NF-κB activity in MM because inhibition of both canonical and non-canonical pathways is necessary to efficiently block total activity [49,51].

### Apoptotic pathway

**Figure 4:**
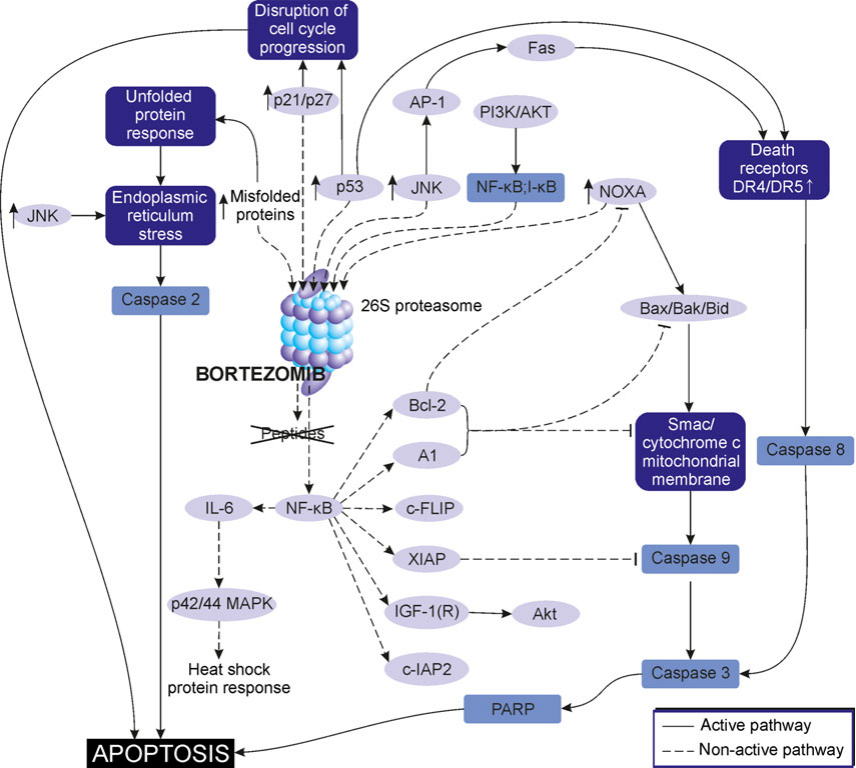
Mechanism of antitumour activity of bortezomib in multiple myeloma (MM) cell. Inhibition of proteasome with bortezomib impairs turnover of multiple proteins resulting in their accumulation in the cell and disruption of multiple signalling pathways within the cell. Consequently, bortezomib-activated signalling pathways lead to disruption of cell cycle and apoptosis.

Inhibition of proteasome promotes programmed cell death of MM cells, as bortezomib is a potent activator of three distinct apoptotic pathways: the intrinsic pathway mediated by caspase-9 activation, the extrinsic pathway mediated by caspase-8 and death receptors (DR) activation and thirdly, activation of ER stress response pathway that involves caspase-2 (Fig.4) [52–55].

In the first case, bortezomib induces Bax (pro-apoptotic member of the Bcl-2 family) accumulation, its translocation from cytosol to mitochondria, conformational change and oligomerization. Such changes lead to inhibition of anti-apoptotic Bcl-2, release of cytochrome c/Smac from mitochondria and activation of caspase-9 [56,57]. Further, it was elucidated that bortezomib induces caspase-dependent apoptosis by promoting up-regulation of NOXA (pro-apoptotic BH3 member of Bcl-2 family), and down-regulation of apoptosis inhibitors, such as XIAP, Bcl-2 or c-FLIP *via* NF-κB blockade [58]. Bortezomib-induced cell death is also linked to the accumulation of ASF1B, Myc, ODC1, BNIP3, Gadd45α, p-SMC1A, SREBF1 and p53 [59]. Also, bortezomib induces p53-dependent apoptosis in MM cells, as it activates and stabilizes the tumour suppressor p53 protein *via* cleavage of the ubiquitin-ligation enzyme MDM2. Another mechanism of bortezomib-mediated apoptosis is *via* activation of extrinsic apoptotic pathway, as was demonstrated by an increased activity of c-Jun N-terminal kinase (JNK) and increase in death-inducing receptors Fas and DR5 that further enhanced Fas-mediated signalling and caspase-8 activation [58,60,61]. It was further elucidated that bortezomib activates caspase-2, which is associated with ER stress-initiated apoptosis. As caspase-2 functions upstream rather than downstream of mitochondria, it stimulates release of cytochrome c, changes within mitochondrial membrane and further caspase-9 activation [55].

### Cell cycle and migration

In replicating cells *in vitro*, bortezomib seems to cause cell cycle arrest at the transition of G_2_/M phase, which further leads to apoptosis in inhibited cells [62]. Bortezomib has also been shown to stabilize the cyclin-dependent kinase (CDK) inhibitors, such as p21 and p27 as it inhibits their degradation by proteasome, leading to disruption of cell cycle progression, that eventually cause apoptosis as well [39,61]. This effect is partly mediated through inhibition of the NF-κB pathway [38].

Bortezomib *in vitro* triggered inhibition of VEGF and IL-6 secretion by MM patient-derived endothelial cells (MMECs); therefore, it inhibited function of BM milieu relevant to angiogenesis. The observation was also confirmed using an *in vivo* model [63]. Notably, bortezomib is a potent inhibitor of cell migration, as it is able to decrease caveolin-1 expression and prevent phosphorylation of caveolin-1 in MM cell lines. Caveolin-1 is a protein involved in cell motility or migration in a number of tissues and its activation requires VEGF-triggered thyrosine phosphorylation. As bortezomib also decreases VEGF secretion in the BM microenvironment, it prevents activation of caveolin-1 [64].

Using GEP70 and GEP80 models, Shaughnessy *et al*. found proteasome 26S subunit, non-ATPase4 (*PSMD4*) and two other proteasome genes to be up-regulated by bortezomib but not by immunomodulatory agents, dexamethasone or melphalan. Function of PSMD4 is binding and selecting ubiquitin-conjugates for destruction. Further analysis revealed that expression levels of PSMD4 (mapped to 1q21 region) are highly sensitive to copy number, as patients with high PSMD4 expression had four copies of 1q21 and patients with low expression had two copies. Authors anticipated that observed up-regulation of PSMD4 could be caused by preferential killing of normal PCs with two copies of 1q21, as more than 90% of PCs of MM patients tend to have more than two copies of 1q21. Both higher PSMD4 expression levels and higher 1q21 copy numbers affected clinical outcome adversely [65].

### Effect of bortezomib on MM side population

Bortezomib was also shown to reduce tumourigenicity of MM as it targets the side population (SP) fraction of MM cells. The MM SP cells show high tumourigenic potential and self-renewal capability and are further characterized by up-regulation of polycomb-related genes, such as *EZH2* and *EPC1*. As bortezomib can reduce levels of p-histone H3 and EZH2, it effectively increased apoptosis and induced G2/M arrest in MM SP [66].

### Bortezomib and microRNA

**Figure 5:**
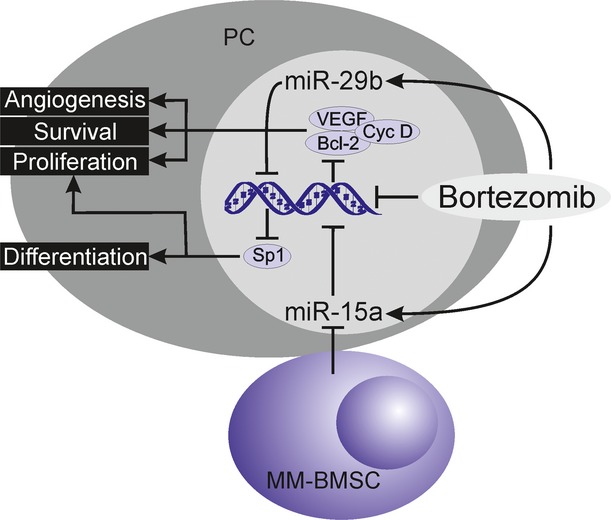
Effect of bortezomib on miR-15a and miR-29b. Bortezomib up-regulates expression of miR-15a and miR-29b, which supports bortezomib-mediated effects on cell differentiation, proliferation and survival. However, expression of miR-15a is suppressed by bone marrow stromal cells (BMSCs) that protect multiple myeloma (MM) cells from bortezomib-induced apoptosis, as they block repression of bortezomib downstream targets (VEGF, cyclin D, Bcl-2-B-cell lymphoma, Sp1–transcription factor Specificity protein 1).

It was shown *in vitro* that bortezomib influences also microRNA (miRNA) expression, as the treatment of MM cell lines with bortezomib led to a dose-dependent increase in apoptotic cells and up-regulation of miR-29b. In this study, enforced expression of miR-29b also strongly increased bortezomib-induced growth inhibition, and thus potentiated its anti-MM activity. Moreover, phosphatidylinositol-3-kinase (PI3K)/AKT pathway played a major role in the regulation of miR-29b-Sp1 (transcription factor Specificity protein 1) loop and induction of apoptosis in MM cells [67]. Two other miRNAs, miR-15a and miR-16, are down-regulated in primary MM cells, their expression inversely correlated with the expression of VEGF and their ectopic overexpression *in vivo* resulted in inhibition of tumour growth and angiogenesis [68]. *In vitro* treatment of MM cells by bortezomib led to up-regulation of miR-15a in MM cells, although it was inhibited by MM-BMSCs. Interestingly, as MM-BMSCs are able to suppress miR-15a expression, they provide survival support and protect MM cells from bortezomib-induced apoptosis, as they block repression of bortezomib downstream targets (VEGF, cyclin D, Bcl-2; Fig.5) [69].

### Further effects

Bortezomib prevents repair of damaged DNA, reduces adhesion of MM cells to BM cells by inhibiting the mitogen-activated protein kinase (MAPK) signalling pathway and inhibits tumour angiogenesis [39,70]. Angiogenesis in the BM environment plays an important role in MM pathogenesis and disease progression. It was shown in pre-clinical models using MMECs that bortezomib inhibited cell proliferation, chemotaxis, adhesion and capillary formation, which further supported its angiogenic inhibitory activity *in vivo*. Furthermore, it inhibited the expression and secretion of several pro-angiogenic factors, including VEGF [63].

Bortezomib also participates in osteoclasts apoptosis and osteoblasts differentiation. It was elucidated that it promotes matrix mineralization and calcium deposition by osteoprogenitor cells and primary mesenchymal stem cells (MSCs) *via* Wnt-independent activation of β-catenin/TCF (transcription factor) signalling and nuclear accumulation of β-catenin. Both these factors are required for promoting MSCs differentiation into osteoblasts [71].

## Second-generation of proteasome inhibitors

The phenomenal success of bortezomib in MM treatment increased the interest of scientific community in PIs. Optimization of the doses of bortezomib and its combination with other anticancer therapeutics reduced its negative side effects and partially suppressed resistance. Further, new generation of PIs was developed and was expected to bring even better results. Carfilzomib, ixazomib and marizomib represent the second-generation PIs and offer many benefits in terms of increased overall effectiveness, reduced negative off-target effects and overcoming resistance to bortezomib because of their different chemical structure, biological properties, mechanism of action, irreversibility/reversibility of proteasome inhibition and usage [72].

### Carfilzomib

Carfilzomib, an irreversible inhibitor of proteasome (also known as PR-171, Kyprolis; Onyx Pharmaceuticals, San Francisco, CA, USA), is a tetrapeptide epoxyketone with molecular formula C40H57N5O7 (Fig.3B). Peptide epoxyketones are the most advanced and specific PIs known to date, as were shown in pre-clinical studies, where carfilzomib targeted haematological-specific immunoproteasome. Its promising characteristics and potential to overcome drug resistance led in 2012 to its approval by FDA for MM treatment (www.fda.gov).

#### Mechanism of action

Carfilzomib binds to CT-L (β5) catalytic subunit of the proteasome or LMP7 subunit of immunoproteasome with higher selectivity than bortezomib [73]. In this case, inhibition is an irreversible process that decreases proteasomal activity to less than 20%; therefore, restoration of proteasome activity in cells is only possible by new synthesis of individual subunits and their further compilation into new proteasome [74].

Carfilzomib forms a unique six-atom ring structure with β5 subunit leading to intramolecular cyclization and morpholino adduction. This intermolecular cyclization is a two-step mechanism. In the first step, oxygen from hydroxyl group of Thr1 nucleophilically attacks carbon of epoxyketone, which subsequently leads to formation of hemiacetal. The second step is a nucleophilic attack of the α-amino nitrogen of Thr1 to C2 carbon-epoxide ring, resulting in the formation of the morpholine adduct [75,76].

Compared to bortezomib, carfilzomib has only little off-target activity outside the proteasome, can induce apoptosis of bortezomib-naïve and even bortezomib-pre-treated MM cells without increased toxicity and is also more effective in xenograft models, which is consistent with its higher affinity for the proteasome [74,77].

In MM cells exposed to carfilzomib, induction of both external and internal apoptotic cascade was observed, with significant elevations of caspases-3, -7, -8 and -9. Programmed cell death has been associated with activation of JNK, mitochondrial membrane depolarization and cytochrome c release. Moreover, an initial decrease in phosphorylated eIF2 was observed, in connection with the ER stress that is induced by accumulation of non-functional proteins and increased levels of NOXA (pro-apoptotic member of Bcl-2 family) [15,74].

Recently, it has been elucidated that carfilzomib promotes MSCs differentiation into osteoblasts with a mechanism similar to that used by bortezomib. It was also shown that carfilzomib does not affect β-catenin gene expression, implying that it induces activation of β-catenin/TCF activity by blocking β-catenin degradation [78].

### Ixazomib

Ixazomib (MLN9708, Takeda/Millenium Pharmaceuticals), an analogue of boric acid, is the first orally administered, reversible PI, which has demonstrated greater potential activity against MM cells than bortezomib in *in vivo* pre-clinical studies [79]. This second-generation PI with chemical formula C20H23BCl2N2O9 is immediately hydrolyed in aqueous solution or plasma to MLN2238, a biologically active form (Fig.3C) [80]. Therefore, it is capable of a wider distribution in blood in a stable form and has greater pharmacodynamic effects in tissues [81].

#### Mechanism of action

Ixazomib (its active form MLN2238), just like bortezomib, inhibits particularly the CT-L (β5) subunit of the 20S proteasome. Moreover, in higher concentrations, it is able to inhibit C-L (β1) and T-L subunit (β2) and induce accumulation of ubiquitinated proteins [79,82]. It has a shorter 20S proteasome dissociation half-life than bortezomib and an improved pharmacokinetic and pharmacodynamic profile. Both ixazomib and bortezomib showed time-dependent reversible proteasome inhibition; however, proteasome dissociation half-life for ixazomib was determined to be about 6-fold faster than that of bortezomib (half-life of 18 and 110 min. respectively) [82].

Ixazomib is responsible for caspase-dependent induction of apoptosis and inhibition of cell cycle in MM cells. Administration of the drug leads to activation of caspase-8, -9 and -3, increased levels of p53, p21, pro-apoptotic proteins NOXA, PUMA, transcription factor E2F and *vice versa* reduced levels of cyclin D1 and CDK6. Treatment with ixazomib also induced expression of Bip and CHOP – heat shock protein and transcription factor connected with ER, which expression is induced by cellular stress and is involved in mediating apoptosis. Further, ixazomib effectively inhibits the canonical and non-canonical NF-κB pathways in MM supporting cells, thus influencing cytokines important for growth and survival of MM cells secreted by BMSCs. In this way, cyto-protective effects of BM microenvironment on MM cells are disrupted. It was also shown that ixazomib inhibits tumour-associated angiogenic activity, as the number of VEGFR2- and PECAM-positive cells (cells containing two distinct markers of angiogenesis) was reduced [79]. Study on mouse models revealed that unlike bortezomib, ixazomib possibly relieves bone osteolysis, the most common symptom of MM [80].

MicroRNA profiling of MM cells treated with ixazomib showed increased expression of miR-33b. Increased expression of this miRNA is associated with reduced migration and viability of MM cells as well as with increased apoptosis and sensitivity of MM to ixazomib. Moreover, overexpression of miR-33b led to negative regulation of oncogene PIM-1. Therefore, Tian *et al*. proposed that miR-33b acts as a tumour suppressor which is involved in the apoptosis of MM cells induced by ixazomib treatment, leading to inhibition of tumour growth and increased survival of human MM xenograft models [83].

### Marizomib

Marizomib, also known as NPI-0052 or Salinosporamid A (Nereus Pharmaceuticals), is a secondary metabolite of obligate marine bacterium, actinomycetes *Salinispora tropica*; it is the first natural PI, which has been included in MM clinical research [84]. Chemically, marizomib is bicycle β-lactone-γ-lactam with molecular formula C15H20ClNO4 (Fig.3D). Unlike all others PIs, it does not contain a peptide chain in its structure; therefore, it is structurally distinct from bortezomib and carfilzomib. In pre-clinical studies with MM cell lines, marizomib was shown to be highly effective [85]. The combination of bortezomib with marizomib would allow using individual drugs in such concentrations that are non-toxic for patients and improve combined anti-myeloma effect of drugs [86].

#### Mechanism of action

Unlike bortezomib and carfilzomib, which are selective for the CT-L activity of the proteasome, marizomib inhibits all three enzymatic activities of the proteasome as it binds irreversibly with high affinity to the CT-L (β5) and T-L (β2) catalytic sites as well as with lower affinity to the C-L (β1) subunit [87]. It contains a β-lactone ring that is uniquely substituted with a chloroethyl group playing a role in its irreversible properties. This group binds to the S2 binding pocket of the active site, and as chlorine behaves as a leaving group, it is eliminated to render a stable cyclic ether end product following acylation of the catalytic enzyme active site Thr1O^γ^ by the β-lactone of the inhibitor [42].

Comparably to bortezomib, marizomib inhibits the canonical NF-κB pathway and related secretion pathways, such as IL-6, TNF-α and IL-1β. [87]. On the other hand, unlike bortezomib, which activates both caspase-8 and -9, the apoptotic effect of marizomib is mainly mediated by caspase-8 activation and, to a lesser extent, by caspase-9. As the mechanism of caspases activation by marizomib is different than in bortezomib-mediated activation and relies primarily on caspase-8 activation, it allows to overcome the resistance of MM cells to apoptosis also with Bcl-2 mutations, leading to overexpression of Bcl-2. It was shown that overexpression of Bcl-2 in MM cells confers drug resistance and partially protects MM cells against bortezomib; however, caspase-9 activation by marizomib is minimally affected by Bcl-2 overexpression [87]. Apoptotic signal leads to the release of cytochrome c and Smac proteins from mitochondria to the cytoplasm, generation of oxygen radicals and activation of caspases. Moreover, marizomib is able to induce apoptosis in MM cells even in the presence of MM growth factors IL-6 and insulin growth factor-1 (IGF-1) and is involved in blocking IL-6 secretion in BMSCs without affecting their viability. Notably, marizomib significantly blocks MM cells migration induced by VEGF and thus confirms its anti-angiogenic effect [87].

## Novel proteasome inhibitors

### Oprozomib

Oprozomib (ONX0912; Onyx Pharmaceuticals), a new orally bioavailable and selective peptide epoxyketone PI, represents a derivate of carfilzomib, which irreversibly inhibits proteasome resulting in longer duration of inhibition compared with bortezomib (Fig.3E). Just like bortezomib and carfilzomib, oprozomib is highly selective for CT-L (β5) subunit of proteasome; however, in contrast with bortezomib, it specifically inhibits only N-terminal threonine active proteasome subunits [88].

The orally bioavailable oprozomib inhibits proteasome with the same efficacy as intravenously delivered carfilzomib, although in higher concentrations of the drug. It is able to activate JNK and inhibit NF-κB pathways [88,89]. Further *in vitro* study using MM cell lines showed that oprozomib inhibits growth, migration and induces apoptosis of MM cell lines and its activity is associated with activation of caspase-8, -9 and -3, and poly(ADP) ribose polymerase (PARP). Oprozomib, suchlike carfilzomib, directly inhibits osteoclasts differentiation and function *in vitro*. Conversely, it directly stimulates transforming growth factor-β (TGF-β) and MAPK signalling pathways leading to increased activity of UPR, which results in enhanced osteoblasts differentiation and matrix mineralization. Therefore, in MM, oprozomib in a similar way as carfilzomib, shifts the BM microenvironment from catabolic to anabolic state. Efficacy of oprozomib was also tested *in vivo* using mouse models. Comparably to carfilzomib, oprozomib inhibited MM growth, prolonged survival of mouse models, decreased tumour burden and inhibited bone resorption [31,88]. Moreover, oprozomib in combination with low-dose bortezomib showed a synergistic anti-MM activity. However, mechanisms mediating combined anti-MM activity of both PIs remain to be defined [88].

### Delanzomib

Delanzomib (CEP-18770; Teva Pharmaceuticals, North Wales, PA, USA) is an orally active, reversible, boronic acid-based PI. Suchlike bortezomib, it exhibits high potency primarily against CT-L (β5) and then C-L (β1) activity (Fig.3F).

An *in vitro* study showed that it effectively decreases NF-κB activity and expression of several NF-κB downstream effectors; furthermore, it has strong anti-angiogenic activity and potently represses receptor activator of NF-κB ligand (RANKL)-induced osteoclastogenesis [90]. Further *in vitro* study on MM cell lines compared delanzomib with bortezomib in terms of specificity and activity profiles. While the two PIs show comparable proteasome inhibitory effects on cell lines, *ex vivo* study on pre-clinical mouse model of human MM showed that delanzomib induced an improved response in MM tumours and is active also against bortezomib-resistant cells [91].

Delanzomib was shown in pre-clinical and clinical studies to be effective in combination with melphalan or bortezomib with favourable cytotoxicity profile [92,93]. Although it showed promising effect and favourable toxicity in the initial studies, its further research has been suspended because of unmanageable toxicity (Teva, personal communication).

## Mechanism of resistance and cross-resistance of PIs

Despite high efficacy of bortezomib, there are still MM patients that are primarily resistant or develop secondary resistance to bortezomib during treatment [94]. So far, several molecular mechanisms of resistance development have been identified. One of them is Ala49Thr mutation in the β5 subunit (PSMB5) of the proteasome, which is situated in a binding site for bortezomib and leads to excessive synthesis of PSMB5. Higher levels of PSMB5 at the RNA or protein level were shown to be connected with resistance to bortezomib [95,96]. High-throughput RNA screen of MM cell lines further revealed a panel of genes – their suppression enhanced bortezomib sensitivity. These genes included proteasome subunits α- and β-type (*PSMA5*, *PSMB2*, *PSMB3* and *PSMB7*) as well as *Aurora kinase A*, *CDK5* and modulators of the aggresome pathway. Moreover, the authors confirmed that *CDK5* knockdown sensitized MM cell lines to bortezomib and other PIs [97]. In addition, factors downstream of proteasome enzymatic complex can mediate resistance to bortezomib, as was observed in a study where increased resistance of tumour cells to the treatment correlated with elevated levels of anti-apoptotic proteins from the Bcl-2 family and heat shock proteins Hsp27, Hsp70 and Hsp90 [87,98]. Zhang *et al*. revealed that in bortezomib-adapted cell lines, the treatment continued to inhibit proteasome enzymatic activity. However, it did not lead to induction of UPR and accumulation of pro-apoptotic proteins p53, Mcl-1S and NOXA because the cells displayed increased expression of factors protecting them from bortezomib-mediated ER stress [99]. More recently, other signalling pathways have been described to mediate bortezomib resistance. It was evaluated that interactions between Notch receptors on MM cells and Notch ligand DII expressed on MM-BMSCs could contribute to bortezomib resistance. Activation of Notch signalling leads to up-regulation of CYP1A, a cytochrome P-450 enzyme involved in the metabolism of a variety of xenobiotic compounds [100]. Blockade of Notch pathway and inhibition of CYP1A expression was able to increase sensitivity of MM cells to bortezomib *in vitro* [101]. Further *in vitro* study on MM cell line with no mutation in β5 subunit revealed evidence that increased IGF-1 signalling through enhanced IGF-1 secretion and IGF-1R activation was also associated with resistance to bortezomib [102]. Newly, the focus is on POMP (proteasome maturation protein), which is involved in addition of catalytically active β subunits to the hemiproteasome ring initially formed by structural α subunits. In bortezomib-resistant cell lines the levels of POMP mRNA are enhanced compared to their drug-sensitive counterparts. POMP overexpression, that is influenced by NF erythroid-2 (NRF-2), contributes to PI resistance in MM [103].

It was described that bortezomib-resistant cells display a marked cross-resistance to β5-targeted cytotoxic peptides, but not to other classes of therapeutic drugs, therefore cross-resistance of bortezomib-resistant cells is restricted to (peptide) drugs that primarily target the proteasome β5-subunit [96]. Therefore, drugs with similar mechanism of action as bortezomib, such as ixazomib and delanzomib, will unlikely overcome bortezomib resistance. However, new drugs that are based on epoxyketone pharmacophore, such as carfilzomib or oprozomib, differ in terms of their chemical structure and mechanism of action. Moreover, carfilzomib is a more selective inhibitor of the CT-L activity of proteasome and immunoproteasome and shows prolonged irreversible inhibition of proteasome – thus it could overcome resistance to bortezomib [77,104]. In fact, carfilzomib was shown in pre-clinical experiments to overcome bortezomib resistance [74]. Nevertheless, new study using mouse MM model and gene expression profiling revealed that bortezomib-resistant cells show cross-resistance to ixazomib and carfilzomib as well. Results of this study also suggested that resistance to one drug class reprograms resistant clones for increased sensitivity to a distinct class of drugs, such as inhibitors of histone deacetylases [105]. Taken together, the results from pre-clinical studies are contradictory so far; although irreversible PIs demonstrate an ability to overcome some forms of bortezomib-mediated resistance, further studies using *e.g*. combination of irreversible PIs with other chemotherapeutic agents may identify strategies to enhance efficacy or decrease toxic effects. Further, there is a need for additional analyses from currently ongoing studies, which include bortezomib-refractory patients who are treated with either analogues of boric acid PIs, such as ixazomib, or epoxyketones, such as carfilzomib.

## Induction of neuropathy

Peripheral neuropathy (PN) is a significant and most common dose-limiting toxicity of PIs. The pathophysiology and molecular basis of bortezomib-induced PN is not completely understood and current knowledge is limited. Damage of mitochondria and ER seems to play a key role in bortezomib-induced PN genesis, as bortezomib can activate the mitochondrial-based apoptotic pathway [106]. Generally, dipeptide boronates inhibit active proteasome subunits, but also serine proteases. Although inhibition of proteasome by bortezomib has originally been shown to be several orders of magnitude stronger than inhibition of serine proteases [107], it has been revealed that bortezomib inhibits also an ATP-dependent serine protease in mitochondria – HtrA2/Omi. As HtrA2 protects neurons from apoptosis, it is now believed that its inhibition is the cause of PN in MM [108]. In contrast with bortezomib, peptide epoxyketones, such as carfilzomib and oprozomib, specifically inhibit only N-terminal threonine active proteasome subunits. This difference may be responsible for the favourable toxicity profiles and relatively low rates of PN associated with epoxyketone PIs.

Further proposed mechanism of bortezomib-induced PN genesis is dysregulation of neutrophins, as bortezomib inhibits activation of NF-κB and thus blocks the transcription of nerve growth factor-mediated neuron survival [109].

A GEP study compared patients with grade 2–4 late-onset bortezomib-induced PN, patients developing early-onset grade 2–4 bortezomib-induced PN and patients who did not develop bortezomib-induced PN. Results suggested that patients with early-onset grade 2–4 bortezomib-induced PN have dysregulated genes involved in control of transcription, apoptosis and AMPK-mediated signalling. Out of them, AMPK-mediated signalling is of particular interest, because this enzyme stimulates the signalling pathways that replenish cellular ATP supplies in response to low glucose, hypoxia, ischaemia or heat shock, which might be triggered in MM cells in response to bortezomib. On the other hand, patients with late-onset grade 2–4 bortezomib-induced PN showed 27 differentially expressed genes when compared to the first group, with the enrichment of expression of genes involved in transcription regulation as well as in the development and function of the nervous system, including *SOD2* and *MYO5A* [110]. Moreover, the authors suggested an interaction between MM-related factors and the patient's genetic background in the development of bortezomib-induced PN, as several of single nucleotide polymorphisms (SNPs) were associated with early-onset bortezomib-induced PN (SNPs located in *caspase 9*, *RDM1*, *ALOX12*, *IGF1R* and *LSM1* genes). In addition, SNPs associated with late-onset bortezomib-induced PN were preferentially located in DNA repair genes (SNPs in *ERCC3*, *ERCC4*, *ATM*, *BRCA1*, *EXO1* and *MRE11A* genes) [110].

Apart of mechanisms mentioned above, another possible mechanism for different rates of PN induced by PIs is their diverse proteasome dissociation half-life, pharmacokinetics and pharmacondynamics (Table3). It was shown that in the group of boronic acid PIs, ixazomib has a six-fold faster proteasome dissociation half-life than bortezomib, greater overall tumour pharmacodynamic effect than bortezomib and prolonged overall survival in a mouse model. All of these features might stand for lower toxicity [82]. Further, as was tested on a fruit fly model, the group of epoxyketone PIs exerts significantly milder impact on neuromusculatory system than bortezomib [111].

**Table 3:** Key features of different proteasome inhibitors

Inhibitor of proteasome	IC 50 for CT-L activity	IC 50 for C-L activity	IC 50 for T-L activity	Half-life (minutes)	Application
Bortezomib	7.9 ± 0.5 nM	53 ± 10 nM	590 ± 67 nM	110	Intravenous
Carfilzomib	<5 nM	2400 nM	3600 nM	<30	Intravenous
Marizomib	3.5 ± 0.3 nM	430 ± 34 nM	28 ± 2 nM	10–15	Intravenous
Ixazomib	3.4 nM	31 nM	3500 nM	18	Oral
Oprozomib	36 nM/82 nM	ND	ND	30–90	Oral
Delanzomib	3.8 nM	ND	ND	ND	Oral

## Proteasome inhibitors in other diseases

Besides MM, bortezomib has been described to be effective in several other lymphoid malignancies; it demonstrated clinical potential in the treatment of mantle cell lymphoma (MCL) and non-Hodgkin lymphoma and is effective also in treatment of newly diagnosed Waldenström macroglobulinaemia (WM) [112–114]. In 2007, FDA granted approval to single-agent bortezomib for the treatment of relapsed/refractory MCL patients [115] and currently it is tested in multiple clinical trials as a component of cytotoxic chemotherapeutic regimens [116]. Further experience with second-generation PIs, such as carfilzomib is limited. Carfilzomib in combination with other drugs was tested in B-cell lymphomas (DLBCL, MCL) to overcome bortezomib resistance with promising results [117]. Also, it is under investigation in WM patients. Although the data are still immature, the biggest advantage of this drug seems to be the lack of neurotoxicity [118].

## Conclusion

The inhibition of proteasome has become an impressively successful strategy in MM treatment for the past 10 years, because of the particular sensitivity of MM cells to the mechanism of action of such agents. Today, therapies based on bortezomib belong to the standards of care for both relapsed/refractory and previously untreated MM patients, dramatically improving outcome of these patients. Although the exact mechanisms of action of PIs are not yet fully defined, four major mediators of direct anti-MM activity have been identified: transcription factor NF-κB, pro- and anti-apoptotic factors, p53 protein and UPR leading to ER stress response. However, the effect of PIs should be understood as a complex process involving many signalling pathways as neither the inhibition of NF-κB nor inactivating mutations of p53 are able to evoke apoptosis of MM cells induced by PIs. Detailed studies of mechanism of PIs action have a great potential for the discovery of possible molecular targets of new-generation drugs. Continuing development of new-generation PIs will likely offer further opportunities and better regimens in terms of treatment efficacy, acceptable tolerability, administration and quality of life of MM patients. Nowadays, PIs are the most effective group of anti-MM drugs, and they will surely be a cornerstone in all combination regimens used in MM treatment even in the future.
